# Experimental study on dimensional variations of 3D printed dental models based on printing orientation

**DOI:** 10.1002/ccr3.8630

**Published:** 2024-03-05

**Authors:** Paula Perlea, Cosmin Stefanescu, Madalina‐Georgiana Dalaban, Alexandru‐Eugen Petre

**Affiliations:** ^1^ Department of Endodontics Carol Davila University of Medicine and Pharmacy Bucharest Romania; ^2^ Department of Prosthodontics Carol Davila University of Medicine and Pharmacy Bucharest Romania; ^3^ Department of Orthodontics Carol Davila University of Medicine and Pharmacy Bucharest Romania

**Keywords:** 3D print, accuracy, dental model, resin, software, trueness

## Abstract

This research investigates the trueness and precision of 3D printing technology in dental applications, specifically focusing on dimensional variations observed in models printed at different angles. The methodology involved importing a dental model into slicing software, adjusting its orientation, and implementing support structures for stability. Subsequently, the model underwent 3D printing five times for each orientation using appropriate equipment and underwent post‐processing steps, including cleaning, washing, and UV‐light post‐curing. The printed models were then scanned using a specialized desktop scanner for further analysis. Accuracy assessment was carried out using dedicated software, employing an algorithm for precise alignment by comparing the scanned files. Color deviation maps were utilized to visually represent variations, aiming to evaluate how positioning during printing influences the trueness and precision of 3D‐printed dental models. Trueness and precision analyses involved the Shapiro–Wilk test for normality and a one‐way ANOVA to compare means of three independent groups, with statistical analyses conducted using IBM SPSS Statistics software. The color maps derived from 3D comparisons revealed positive and negative deviations, represented by distinct colors. Comparative results indicated that models positioned at 0° exhibited the least dimensional deviation, whereas those at 90° showed the highest. Regarding precision, models printed at 0° demonstrated the highest reproducibility, while those at 15° exhibited the lowest. Based on the desired level of precision, it is recommended that printed models be produced at an inclination angle of 0°.

## 1 INTRODUCTION

The initiation of 3D printing took place with Charles Hull's introduction of stereolithography (SLA) technology in 1980. The continuous advancement of this technology led to notable progress, prompting Charles Hull to establish 3D Systems in 1986, thereby contributing significantly to the scientific evolution of three‐dimensional printing. SLA, in particular, received patent approval from the United States Patent and Trademark Office (USPTO) in August 1984 and was officially granted in 1986, marking a pivotal milestone in the historical trajectory of 3D printing.^1^ Subsequently, SLA technology has undergone continuous evolution, emerging as the predominant form of three‐dimensional printing.^2^ In this procedure, the substrate is immersed in a photosensitive resin.^3^ Utilizing a laser, cross‐sectional outlines of the object are delineated to construct individual layers. Upon complete polymerization of a resin layer, the substrate ascends vertically by a distance corresponding to a single layer thickness, enabling the generation of successive layers. This iterative process is executed numerous times, ranging from hundreds to thousands, resulting in the production of the three‐dimensional object.^2^ The thickness of the polymerizable layer is determined by the specifications of the printer model, ranging from 15 μm to 150 μm. Additionally, the wavelength range of the UV laser employed for polymerizing the photosensitive material is printer‐type dependent, commonly commencing at 200 nm and extending up to 500 nm.^3^

Thermoplastic Extrusion Modeling, also known as Fused Deposition Modeling (FDM), constitutes an alternative printing approach based on the extrusion of a thermoplastic material. In this process, a plastic filament is guided through an extruder, undergoing heating to its melting point, followed by the deposition of layers. Simultaneously, the extruder executes horizontal movements, and the platform ascends vertically after the deposition of each new layer.^3^ The layers deposited undergo thermal bonding or fusion facilitated by chemical agents.^4^

Digital Light Processing (DLP), pioneered by Larry Hornbeck at Texas Instruments in 1987,^5^ shares similarities with SLA and is classified by ASTM (American Society for Testing and Materials) within the same additive manufacturing technology category. It relies on UV light for the polymerization of photosensitive resins.^3^ The distinguishing factor between these technologies lies in the light source, with DLP utilizing a high‐definition projector capable of simultaneously photopolymerizing the resin layer in the x‐y axis.^2^, ^6^ Recognized for its superior speed and efficiency in comparison to SLA,^6^ the DLP technique produces components known for their elevated precision and superior surface finish.^7^

Technological advancements arising from the integration of three‐dimensional printing technology have demonstrated notable impact across diverse domains such as medicine, automotive manufacturing, mechanical engineering, and the arts. Within the realm of dental applications, rapid prototyping stands out as a particularly efficient tool for three‐dimensional printing intricate anatomical structures.^8^ In the field of dental medicine, additive manufacturing technology finds applications in prosthodontics, surgery, orthodontics, endodontics, and tissue engineering.^9^, ^10^

Among the initial applications of additive manufacturing technology in dental medicine was the utilization of digital impressions for the generation of printed models, serving diagnostic purposes, and facilitating the creation of functional models for fixed prosthodontic restorations.^3^ Printed models offer a viable alternative to cast models, exhibiting reduced susceptibility to damage in standard environmental conditions, or during storage.^8^ Three‐dimensional printed models exhibit a level of precision considered clinically acceptable.^11^ A comparative analysis assessing the reproducibility and accuracy between cast models and digital models, as conducted by Park et al., revealed larger but clinically acceptable dimensional changes in the digital models.^12^

In orthodontics, 3D‐printed models are utilized for diagnosis and treatment planning, demonstrating differences in both reproducibility and accuracy based on the employed printing technology. The precision of 3D‐printed models has been substantiated with DLP technology, whereas FDM technology has exhibited geometric imprecision.^13^ Comprehensive records of dental arches undergo processing using specialized software, facilitating virtual simulations of orthodontic treatment. Subsequently, personalized dental appliances, aligners, brackets, and archwires can be manufactured.^13^

Given the necessity for precision and trueness in dental three‐dimensional printing, the existing literature contains information highlighting the impact of printing methodologies on model accuracy. Consequently, a thesis has been proposed to examine the impact of build orientation on the fabrication of DLP‐printed dental models. Thus, the null hypothesis posited was that the build orientation would not have any impact on the precision and trueness of the 3D printed dental models, and there is no difference among group means.

## 2 CASE PRESENTATION

**FIGURE 1:**
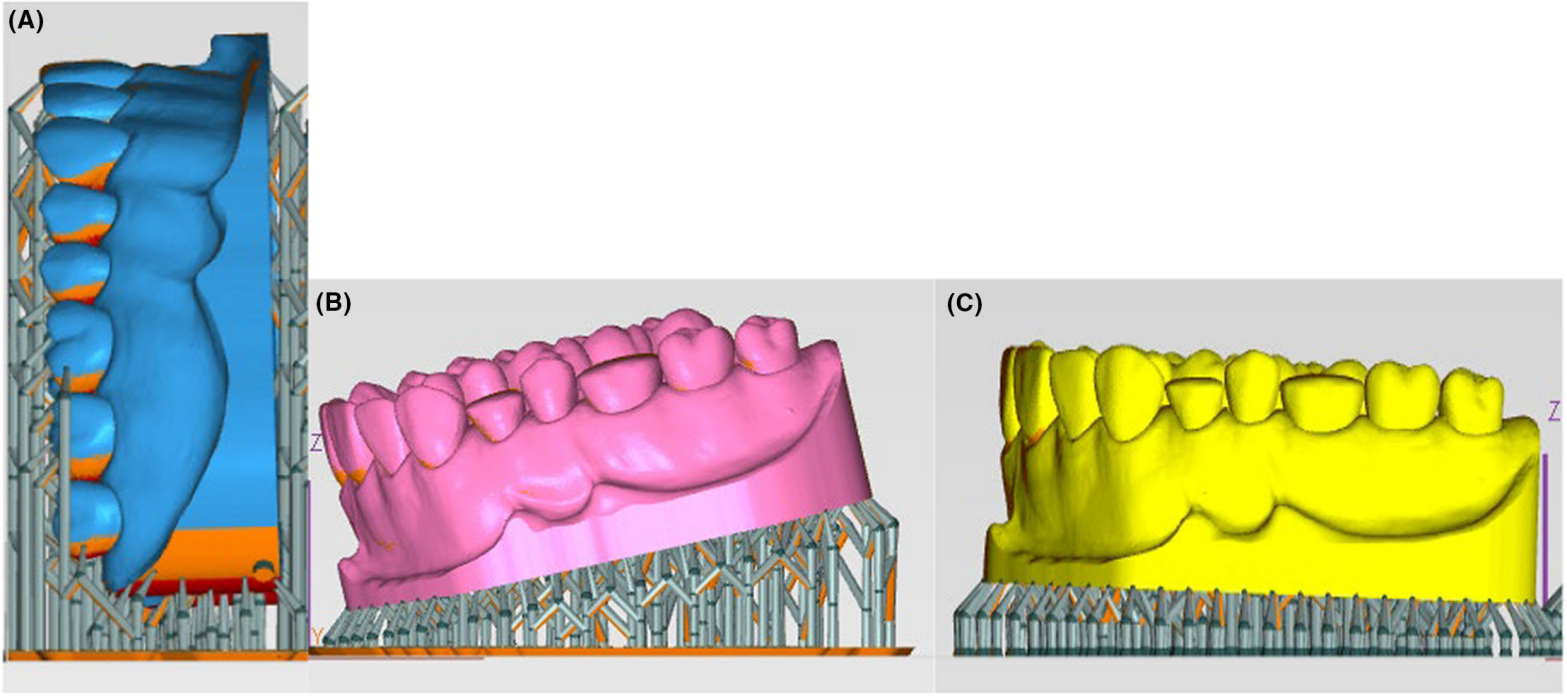
Orientation of the model: (A) 90°; (B) 15°; (C) 0°.

For the experimental study, a 3D image of a dental didactic model (Frasaco GmbH, Germany) was employed. The 3D file (stl) underwent importation into the Phrozen 3D Slice Software (Phrozen, Taiwan) for slicing and subsequent project exportation in ctb format (Autocad Color‐based Plot Style File). The positioning of the model on the printer platform was executed at 0°, 15°, and 90° angles (Figure 1).

**FIGURE 2:**
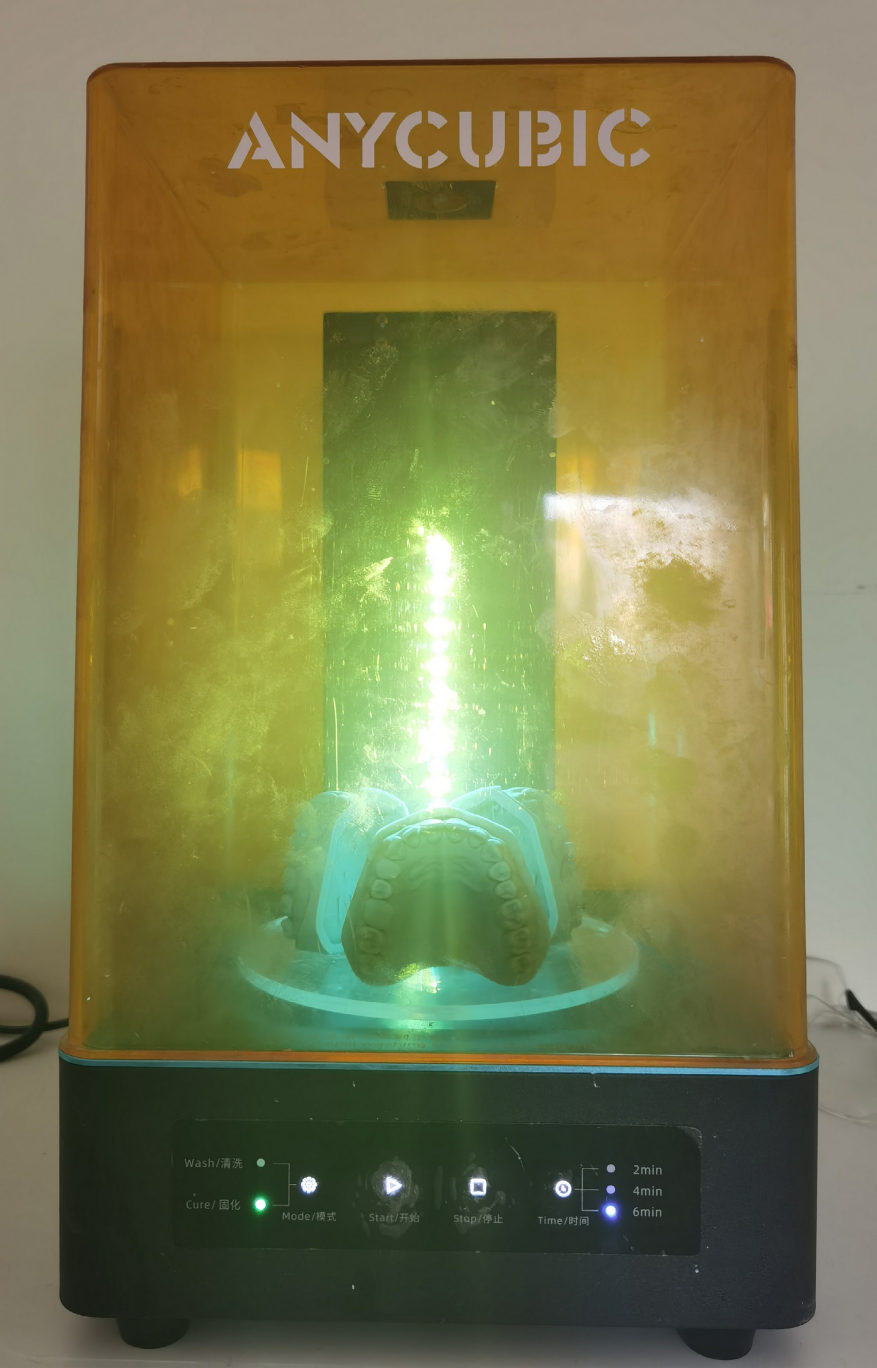
Washing & curing unit from anycubic.

Support structures were automatically generated by the slicing software, with additional manual support structures added in high‐risk areas based on the model's orientation on the platform. The placement of support structures considered the integrity of clinically usable surfaces, particularly the prosthetic area. Utilizing Phrozen Aqua 4 K Resin Gray liquid resin material (Phrozen Technology, Hsinchu, Taiwan), recommended for dental practice due to its low contraction index, the model underwent five printing iterations for each position. The Phrozen Sonic Mini 4 K 3D printer (Phrozen Technology, Hsinchu, Taiwan), employing DLP printing technology with a resolution of 50 μm, was utilized. Models were arranged on the printer platform within the maximum printing volume constraints of 135 × 75 × 130 mm. Post‐printing, the removal of support structures was followed by a two‐step post‐processing procedure involving washing and residue removal in successive isopropyl alcohol (IPA) baths, each lasting 3 min. The second washing step utilized the “Wash” mode of the Anycubic Washing & Curing Machine (ANYCUBIC 3D Printing, Shenzhen, China), adhering to the manufacturer's instructions. (Figure 2).

**FIGURE 3:**
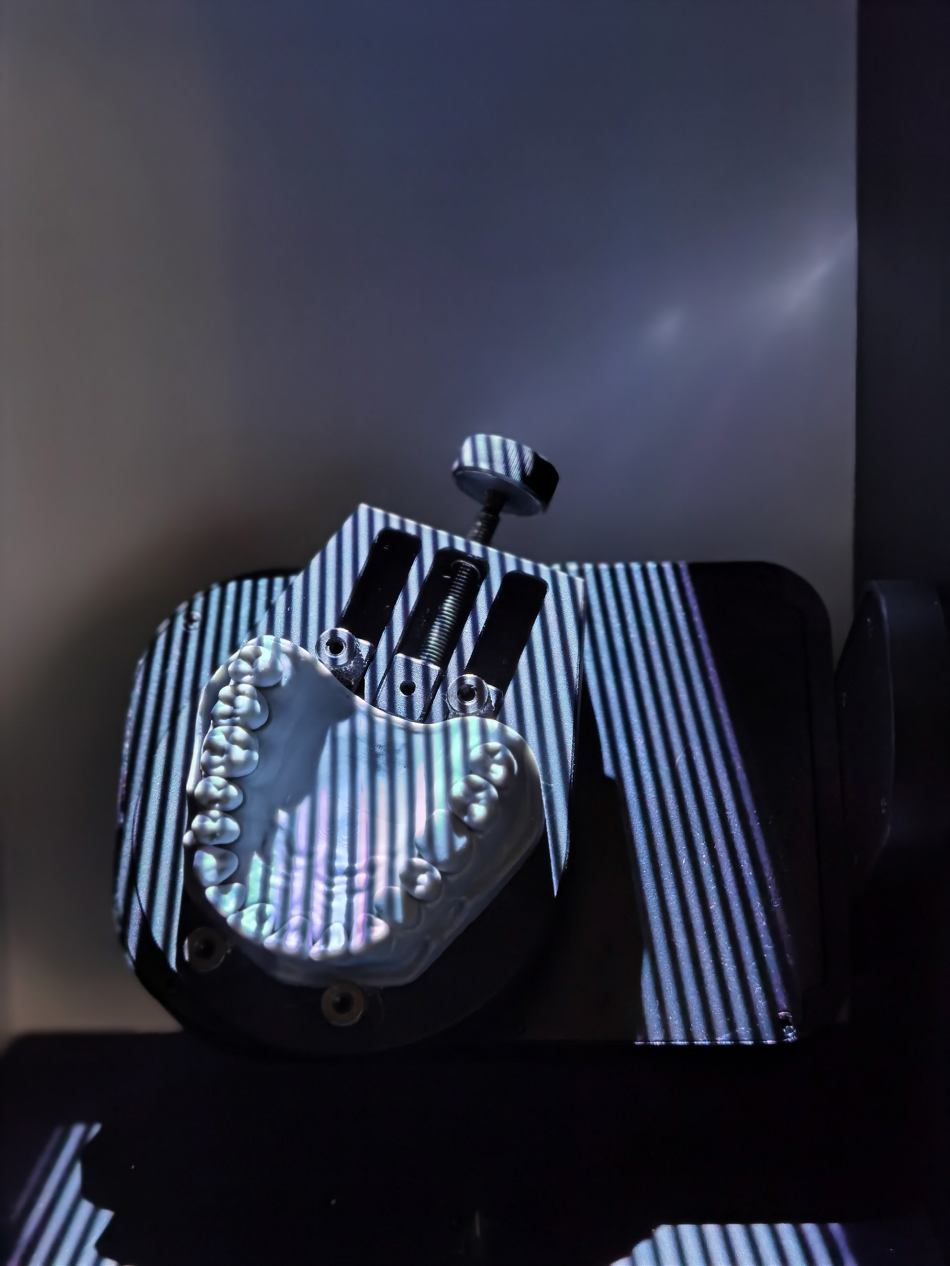
Printed model in the desktop scanner.

Following residue removal through washing, the subsequent steps involve drying and polymerization. The final phase utilized the “Cure” mode of the Anycubic Washing & Curing Machine (ANYCUBIC 3D Printing, Shenzhen, China) for a duration of 30 min. Three‐dimensional images of the models were obtained using a desktop scanner with a resolution of 0.01 mm, Thunk3D DT 300 (Thunk3D Inc., Beijing, China), specifically designed for dental applications (Figure 3). The models obtained through scanning were exported in stl format and subsequently imported into the metrology software.

**FIGURE 4:**
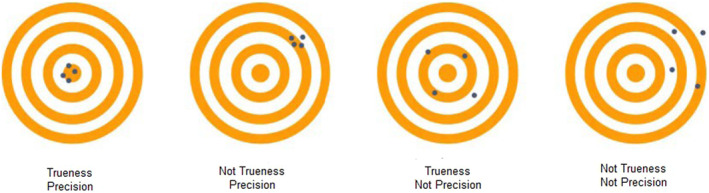
Trueness and precision—representation.

As per the International Organization for Standardization ISO 5725,^14^ accuracy is defined in terms of trueness and precision.^15^ Trueness relates to the minimum distance between the measured test object and the reference object, while precision pertains to the reproducibility of measured values through repeated measurements^16^ (Figure 4).

Another relevant ISO standard in the field of additive manufacturing is ISO/ASTM 52900, which provides a comprehensive classification of this technology into seven distinct process categories: binder jetting, directed energy deposition, material extrusion, material jetting, powder bed fusion, sheet lamination, and vat polymerization (Figure 1). These categories encompass various methods used in additive manufacturing, each with its unique characteristics and applications. Among the most widely utilized technologies in this domain are FDM, laminated object manufacturing (LOM), SLA, selective laser melting (SLM), and selective laser sintering (SLS).^17^

## 3 METHODS

**FIGURE 5:**
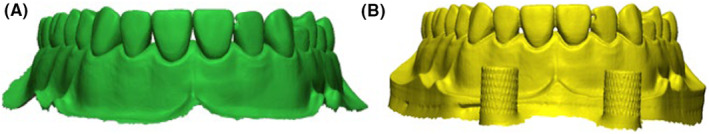
(A) Segmented reference model; (B) scanned test model.

For the analysis of trueness, each stl scan file was indexed with the reference stl file resulting five datasets for each group, while for the analysis of precision, each stl scan file was indexed with each scan of the model in the same orientation category, resulting 10 datasets for each group. For this purpose, Geomagic Control X software (3D Systems, Rock Hill, South Carolina, USA) was used. Geomagic Control X is specialized software designed for the inspection and quality control of three‐dimensional objects, facilitating the processing of 3D scan data for measurement, comparison, and communication of results. This software employs the Iterative Closest Point (ICP) algorithm, a widely utilized method for 3D file registration. The algorithm establishes correspondences between two point cloud areas, determining the minimum distance between them. Subsequently, it compares the values from the test file with those from the reference model.^18^ (Figure 5).

**FIGURE 6:**
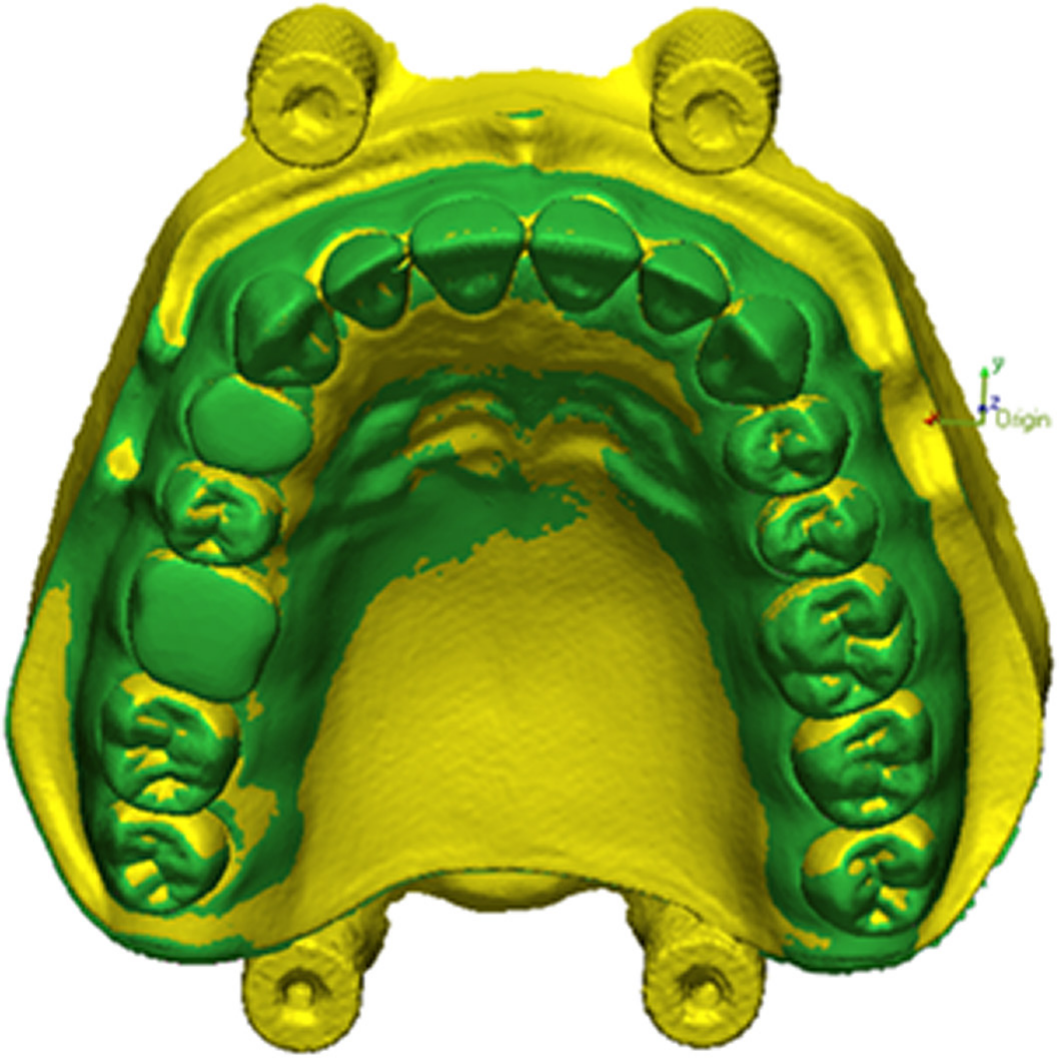
Alignment of best fit models.

The workflow involves importing scanned files in SLA format, with the first file serving as the reference data.^19^ The initial processing of the reference file involves the removal of excess components to obtain minimal information requiring further processing, as the removed parts are no longer involved in the subsequent alignment. Before proceeding with the alignment step, the software incorporates a resegmentation function (Resegmenting Tool), enabling manual selection and division of parts of the model that present additional interest for comparison with the test model.^15^ Utilizing the initial alignment and best fit alignment functions (Figure 6), the models are indexed with standard software precision and subsequently benefit from a superior final alignment compared to the initial alignment.^20^, ^21^

**FIGURE 7:**
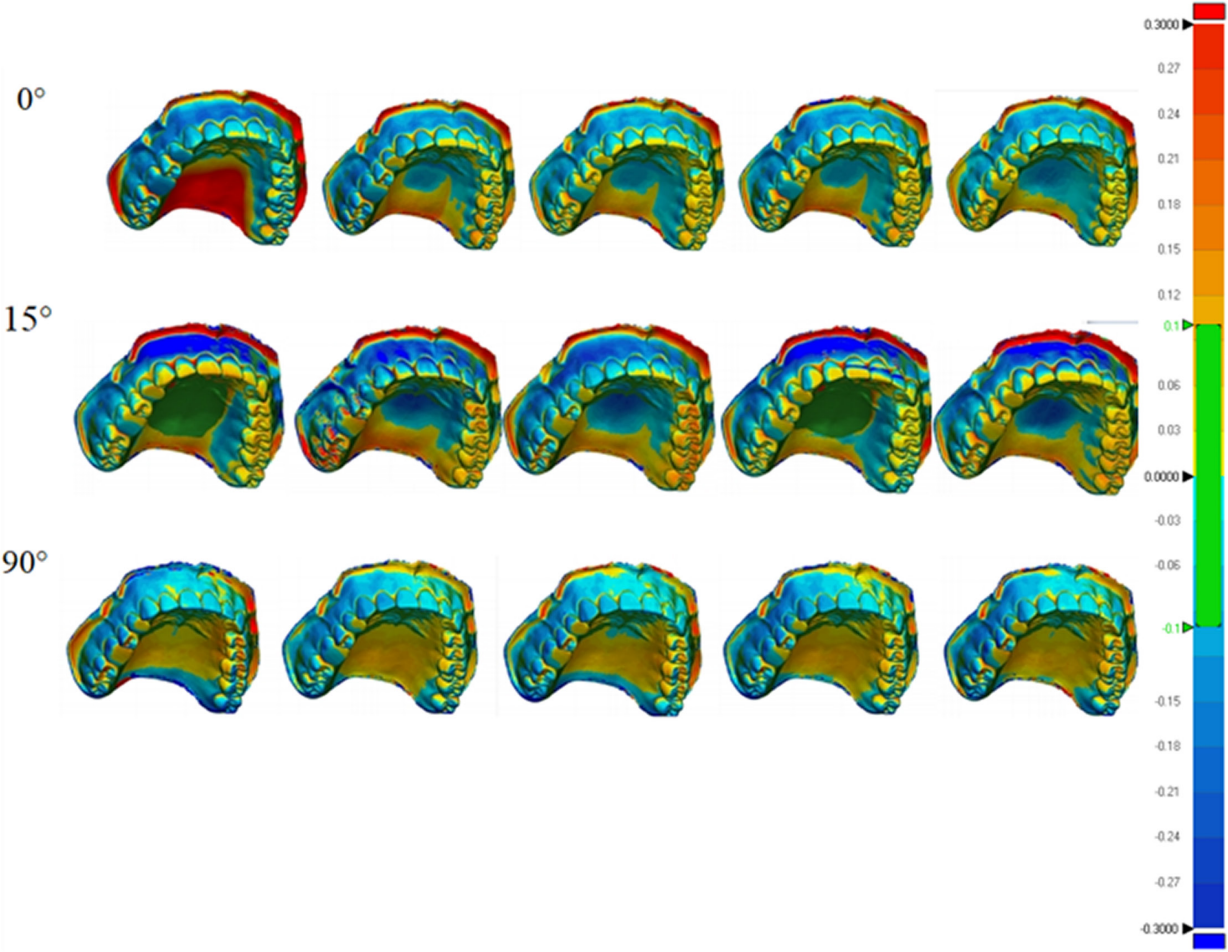
Three‐dimensional evaluation of model trueness.

**FIGURE 8:**
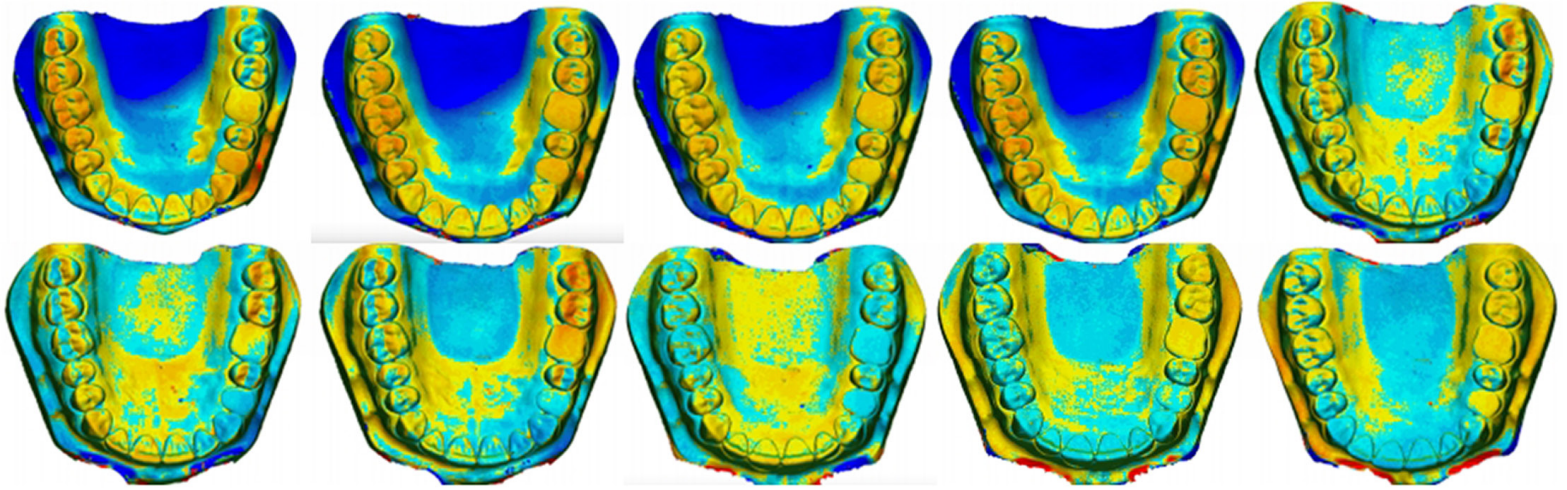
Three‐dimensional analysis of models for precision at 0° position.

**FIGURE 9:**
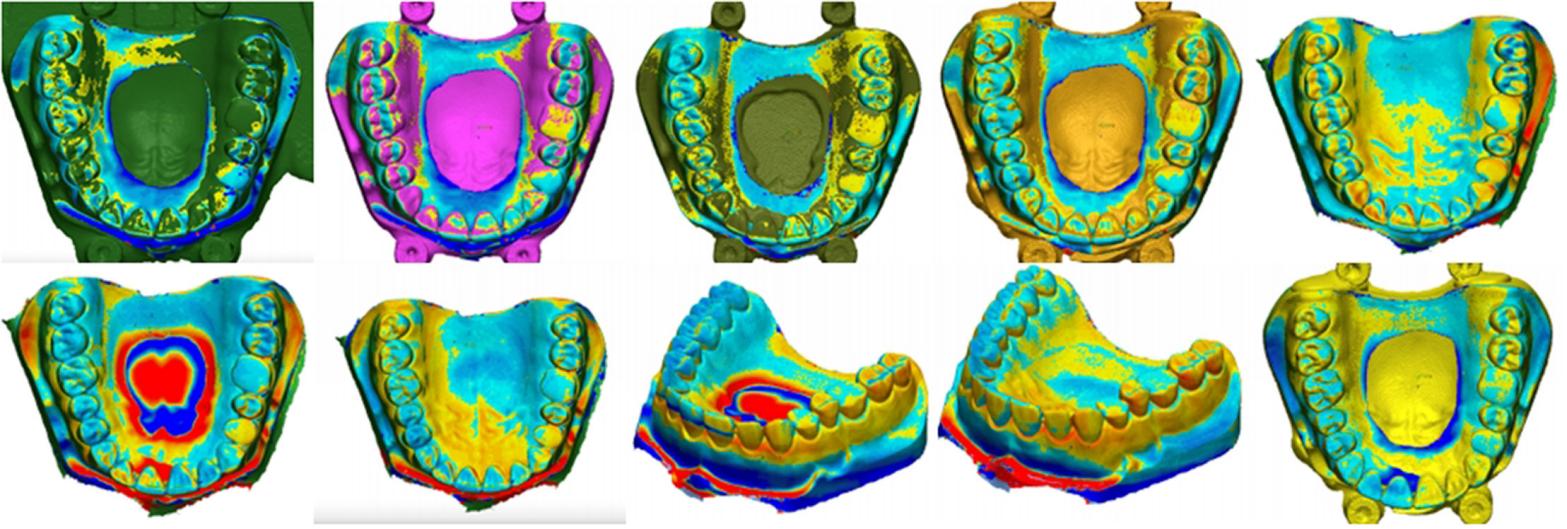
Three‐dimensional analysis of models for precision at 15° position.

**FIGURE 10:**
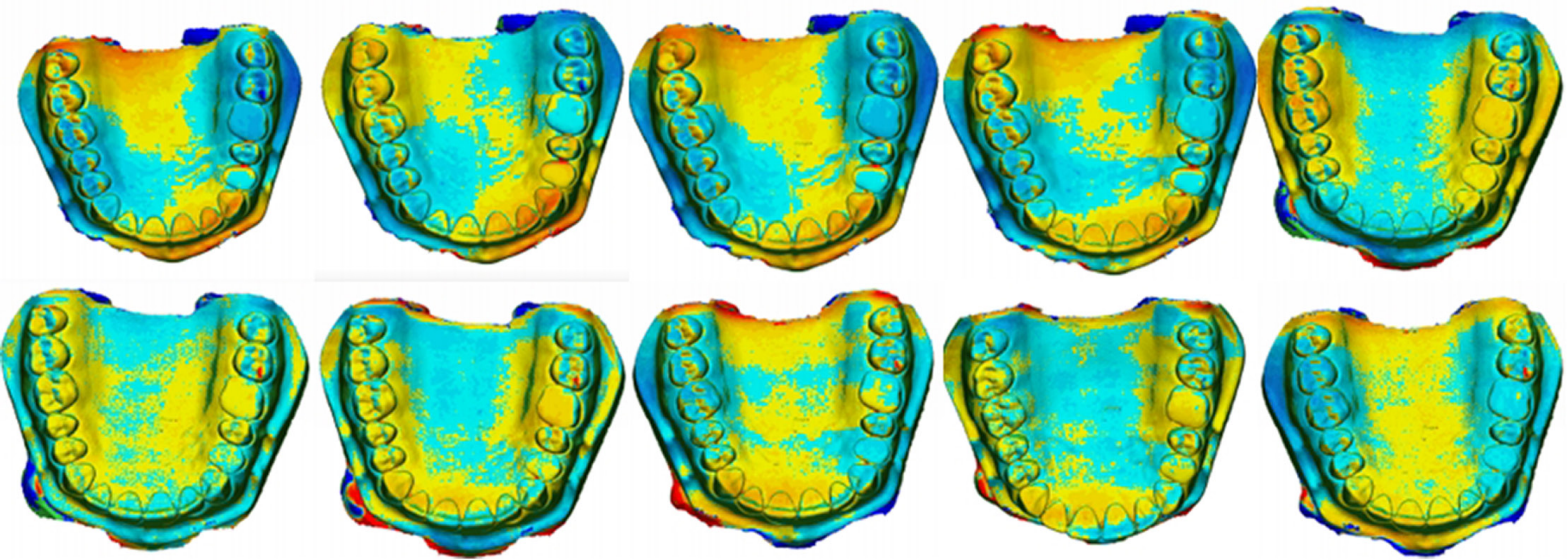
Three‐dimensional analysis of models for precision at 90° position.

The 3D comparison function of the software enables the adjustment of analysis limits and the generation of a color map for analyzing deviations from the reference model. In the color map, green signifies adequate alignment or minimal deviations, dark blue areas indicate deviations below the reference model, and dark red areas indicate positive deviations.^18^, ^22^ A limit of ±300 μm was utilized to obtain the color maps.(Figure 7, Figure 8, Figure 9, Figure 10) Areas displaying deviations beyond the selected limits were colored with dark red and dark blue, while areas between the limits generated variable colors.

The normality of the results was analyzed by Shapiro–Wilk test. Differences were then analyzed by one‐way ANOVA. Significance level was set up to *p* < 0.05. All statistical analyses were performed using IBM SPSS software (IBM, Chicago, IL).

## 4 CONCLUSION AND RESULTS

The color map derived from the 3D comparison for trueness and precision revealed positive deviations depicted by colors ranging from yellow to red, along with negative deviations represented by colors ranging from cyan blue to dark blue.

In terms of trueness, a comparison of results based on minimum and maximum values revealed that models positioned at 0° exhibited the least deviation from the reference model (Table 1), followed by those positioned at 15° (Table 2). Conversely, models positioned at 90° (Table 3) showed the maximum dimensional deviation from the reference model.

**TABLE 1:** Trueness analysis measurements for the 0° position, expressed in millimeters.

Name	Min	Max	Avg	Root mean square	Standard deviation
1	−3.91	3.9102	−0.0444	0.8328	0.8316
2	−3.4056	3.4049	−0.0838	0.5818	0.5757
3	−3.0956	3.0953	−0.0688	0.4723	0.4673
4	−3.1471	3.1467	−0.0798	0.4779	0.4712
5	−3.4445	3.4441	−0.0643	0.5946	0.5946

**TABLE 2:** Trueness analysis measurements for the 15° position, expressed in millimeters.

Name	Min	Max	Avg	Root mean square	Standard deviation
1	−3.9484	3.9479	−0.1495	0.8179	0.8042
2	−3.8327	3.8311	−0.1085	0.7626	0.7548
3	−3.3538	3.3539	−0.0993	0.5659	0.5571
4	−3.7139	3.7136	−0.158	0.7026	0.6846
5	−3.6501	3.65	−0.0862	0.7024	0.6971

**TABLE 3:** Trueness analysis measurements for the 90° position, expressed in millimeters.

Name	Min	Max	Avg	root mean square	Standard deviation
1	−4.2906	4.2899	−0.2158	0.9823	0.9583
2	−3.7173	3.7166	−0.1669	0.7081	0.6881
3	−3.4618	3.4611	−0.1217	0.5987	0.5862
4	−3.6061	3.6054	−0.1622	0.6727	0.6528
5	−3.7285	3.7276	−0.1439	0.7216	0.7071

Concerning precision, an analysis of minimum and maximum values indicated that models printed at a 0° (Table 4) demonstrated the highest dimensional reproducibility, followed by those printed at a 90° (Table 5). Models printed at a 15° (Table 6) exhibited the least data reproducibility.

**TABLE 4:** Precision analysis measurements for the 0° position, expressed in millimeters.

Name	Min	Max	Avg	Root mean square	Standard deviation
1	−3.1419	3.1415	−0.0731	0.4659	0.4601
2	−3.0766	3.0753	−0.0701	0.4686	0.4634
3	−3.14	3.1398	−0.0797	0.4801	0.4734
4	−3.2896	3.2891	−0.0609	0.535	0.5315
5	−2.9041	2.9043	0.0172	0.3536	0.3532
6	−2.9881	2.9878	−0.0031	0.3714	0.3714
7	−3.0633	3.063	0.0223	0.4033	0.4027
8	−2.8349	2.8359	−0.0194	0.3384	0.3379
9	−2.9065	2.9068	−0.0152	0.3691	0.3691
10	−2.8966	2.8955	−0.0067	0.3632	0.3631

**TABLE 5:** Precision analysis measurements for the 90° position, expressed in millimeters.

Name	Min	Max	Avg	Root mean square	Standard deviation
1	−3.1628	3.1627	−0.0418	0.4655	0.4637
2	−3.0866	3.0863	−0.0438	0.4051	0.4027
3	−3.03	3.0301	−0.0541	0.4116	0.4081
4	−3.2039	3.2029	−0.0486	0.494	0.4916
5	−3.1032	3.1031	−0.0332	0.4185	0.4172
6	−3.0624	3.0621	−0.032	0.4064	0.4051
7	−3.1226	3.1229	−0.0148	0.4481	0.4479
8	−3.0798	3.0796	−0.0481	0.4157	0.4129
9	−3.1795	3.1782	−0.0391	0.478	0.4764
10	−2.955	2.9546	−0.0291	0.4004	0.3993

**TABLE 6:** Precision analysis measurements for the 15° position, expressed in millimeters.

Name	Min	Max	Avg	Root mean square	Standard deviation
1	−4.137	4.1367	−0.147	0.8614	0.8487
2	−3.8771	3.8764	−0.1319	0.7336	0.7216
3	−4.0359	4.0359	−0.0859	0.8059	0.8013
4	−3.9272	3.9271	−0.0582	0.77	0.7678
5	−3.2769	3.2767	0.0241	0.4493	0.4486
6	−3.4863	3.4864	−0.0016	0.5355	0.5355
7	−3.5362	3.5361	0.0665	0.5638	0.5599
8	−2.9952	2.9951	−0.0246	0.3976	0.3968
9	−2.9755	2.9759	0.0161	0.4118	0.4115
10	−3.9046	3.9046	−0.0465	0.7784	0.777

**FIGURE 11:**
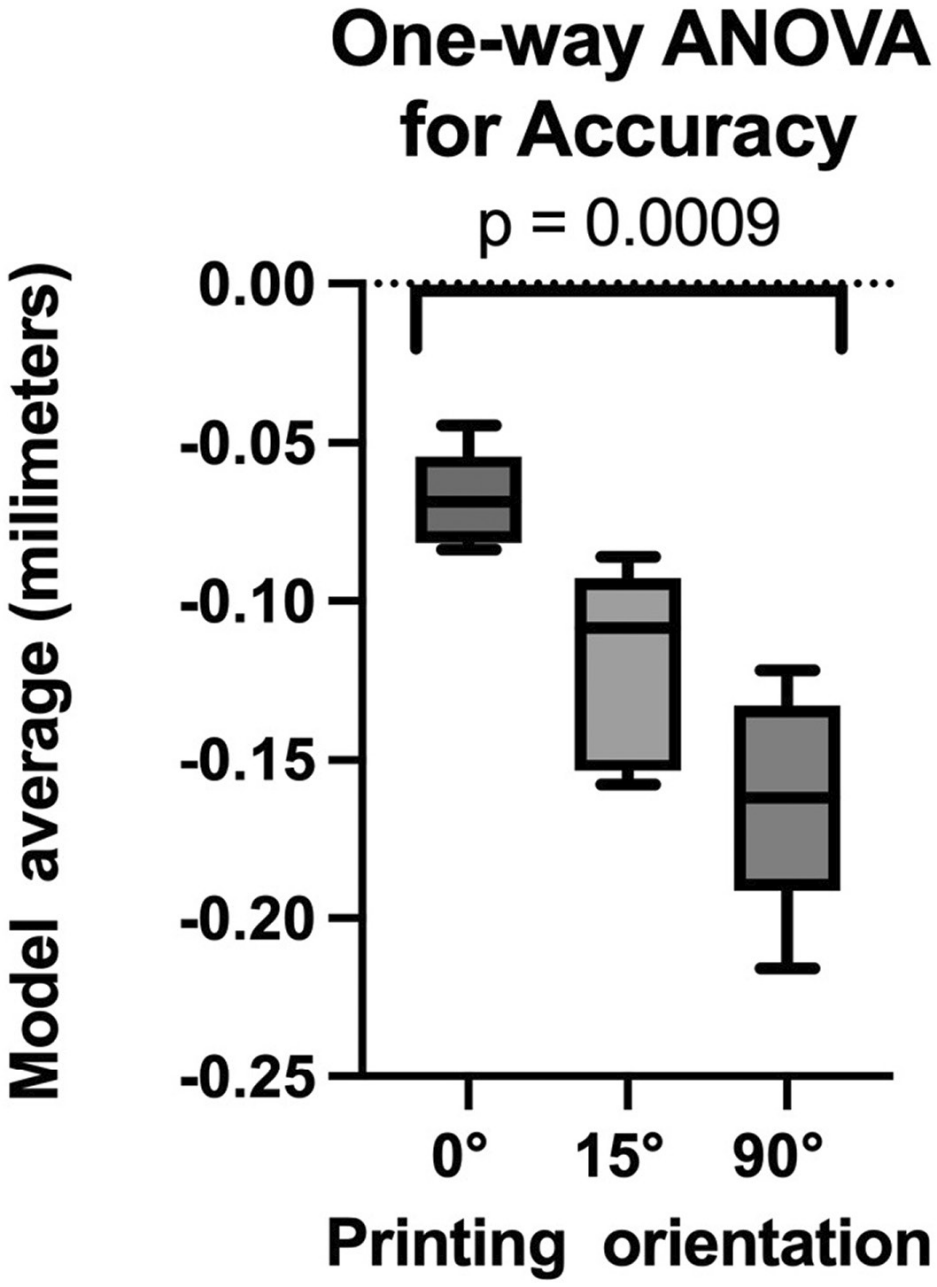
Accuracy.

**FIGURE 12:**
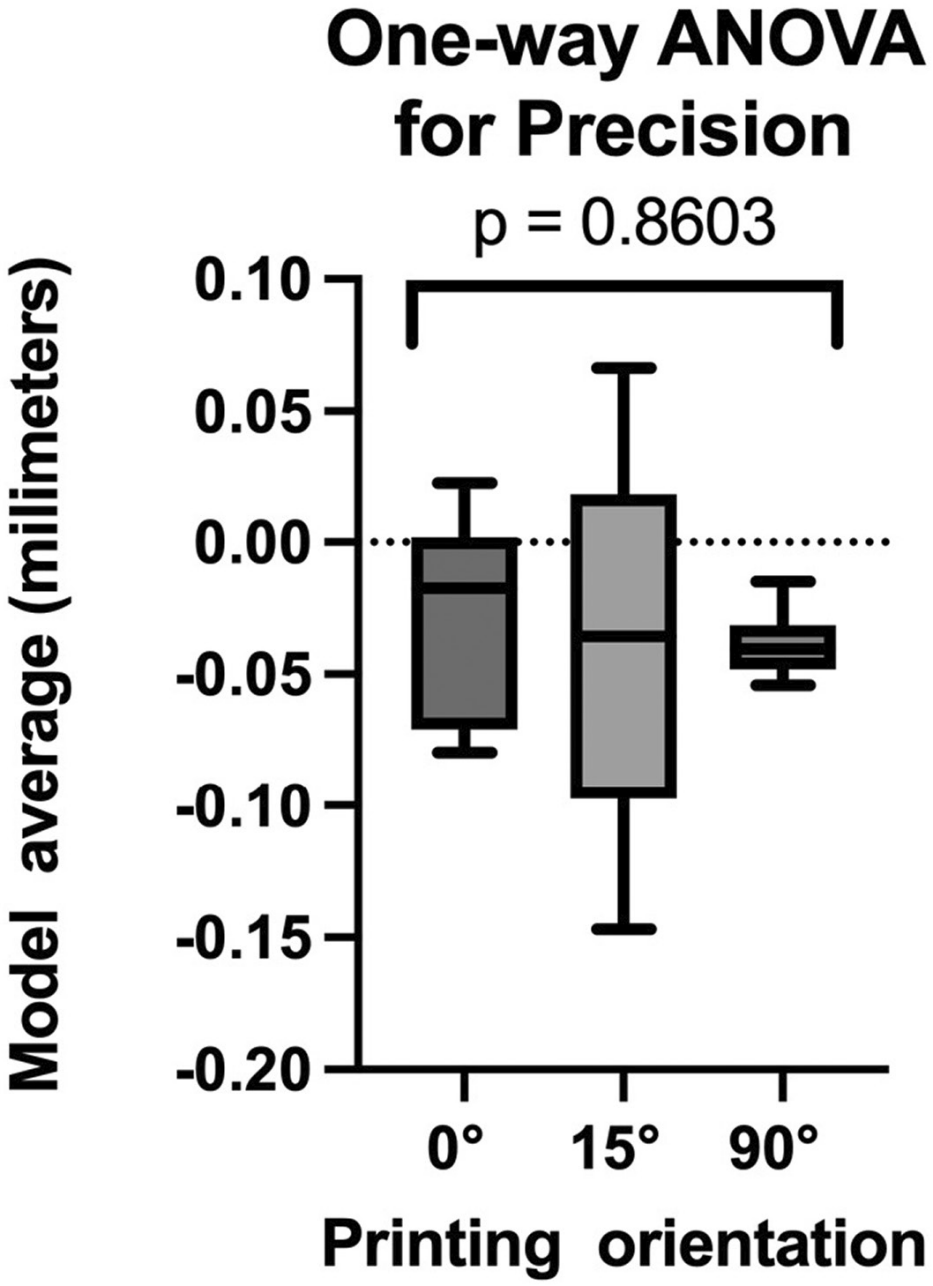
Precision.

The normality of the data was assessed using the Shapiro–Wilk test, which scored *p* > 0.05 for both accuracy and precision. Consequently, one‐way ANOVA was employed for further analysis to explore potential differences in the means of three independent groups. While the test for accuracy resulted in a *p* < 0.05 (Figure 11), the test for precision generated a *p* > 0.05. (Figure 12).

A limitation of this study is that the accuracy and precision of 3D printed models are influenced not only by build orientation. It's crucial to acknowledge that other critical parameters, such as support diameter, layer thickness and support structure connection demand careful consideration in assessing the implications of the findings within the medical context. Another limitation of this study is that the statistical power is not optimal because the limited number of the 3D‐printed models. In consideration of the study's limitations, the following conclusions emerge:

Primarily, the results suggest that printing models at the 0° position yields superior trueness compared to models printed at both 15° and 90° angles, indicating that the 0° position provides an optimal orientation for precise dimensional outcomes. The one‐way ANOVA test demonstrated a significant difference between the three groups with *p* < 0.05.

Additionally, the findings indicate that models printed at the 0° position exhibit the highest level of dimensional reproducibility. Subsequently, models printed at 90° show slightly lower reproducibility, followed by those printed at 15°. The one‐way ANOVA test revealed no significant difference between the three groups with *p* > 0.05.

These conclusions underscore the critical role of print positioning in achieving accurate 3D models. Further investigations are warranted to delve into the contributing factors and refine the printing process, particularly in the context of its application in dental prosthetics.

## 5 DISCUSSION

After conducting the statistical analysis, we observe a significant difference among the three analyzed groups for trueness, rejecting the null hypothesis. In contrast, the statistical analysis conducted for precision did not indicate a significant difference among the three groups. The obtained results offer supporting evidence for the thesis, revealing minimal deviations in the models printed at 0° compared to those at 15° and 90°. Consequently, the study substantiates the validity of the thesis based on the observed trueness analysis of the printed models. A fundamental principle in model orientation on the printing platform is that deviating from 0° reduces the surface area of each layer, leading to decreased contact between the platform and the resin tank. This results in reduced force exerted on the model during the layer‐building process as the printer platform lifts.^23^ The higher deviations observed in models oriented at 15° could be attributed to the manufacturing process. Multiple models (two or five) were placed on the platform, increasing the contact area with the resin tank and leading to larger printed layers per exposure. Support structures were initially auto‐generated at 80% density and manually added in high‐risk areas. Generating support structures is easier for models with flat surfaces compared to textured ones.^24^ The metrology software employed for comparative analysis individually indexed the test models to the reference model. However, in the precision analysis, a preset option for indexing all models was not employed; instead, manual indexing was conducted in pairs following the manual segmentation of each model. Unfortunately, no standardized data reference was considered for these processes.

The study's findings are consistent with prior research emphasizing the significant impact of the presence or absence of a cross‐arch plate and variations in internal structure on the characteristics of 3D printed models using the DLP method. Specifically, Group P, featuring a cross‐arch support plate, demonstrated superior stability compared to Group U, where deviations were noted in model contraction on both lingual sides in the posterior region.^25^

Song et al.^26^ showed that 90° orientation yielded the best accuracy when compared to 0° and 15°. Hussein et al.^27^ concluded that build orientation had an influence on the accuracy of the 3D printed partial denture frameworks, especially at a 135‐degree angle of maxillary design and 150‐degree of mandibular design. Tahir et al.^28^ had a similar conclusion, that the horizontally printed surgical guides exhibited superior accuracy in comparison with those printed at 45° and 90° orientations. Ko et al.^29^ showed that build angle and layer height have statistically significant interactive effects on the accuracy of 3D‐printed dental models. Other authors^30^ concluded that 45° build orientation yielded the most accurate 3D‐printed denture from a MultiJet 3D printer. Suk Shim et al.^31^ demonstrated that specimensprinted at a 90‐degree orientation showed the lowest error rates in comparison with those printed at a 45‐degree orientation. Another study conducted a comparison of various 3D printer technologies for chairside resin model printing, demonstrating their capability to achieve accurate results within 30 microns in each XYZ dimension. The study concluded that these printers are suitable for clinical practice, exhibiting overall errors within clinically acceptable levels of under 100 microns.^32^

Morón‐Conejo et al.^33^ conducted a comparative analysis of the accuracy, trueness, and precision of five different 3D printers utilized for full‐arch models of patients. This included both industrial and dental desktop printers. The findings revealed statistically significant differences, with Multijet printing technology employed in industrial 3D printers exhibiting superior results compared to DLP and SLA technologies used in dental desktop printers. The study emphasized the importance of standardizing the 3D printing protocol and parameters, material usage, postprocessing, and assessment time for accurate performance comparisons in the field of dental 3D printing.

## AUTHOR CONTRIBUTIONS

**Paula Perlea:** Methodology; project administration; supervision; writing – original draft; writing – review and editing. **Cosmin Stefanescu:** Methodology; project administration; supervision; writing – original draft; writing – review and editing. **Madalina Georgiana Dalaban:** Methodology; project administration; supervision; writing – original draft; writing – review and editing. **Alexandru Eugen Petre:** Methodology; project administration; supervision; writing – original draft; writing – review and editing.

## ETHICS STATEMENT

This study was conducted in accordance with the declaration of Helsinki.

## CONSENT STATEMENT

Written informed consent was obtained from the patient to publish this report in accordance with the journal's patient consent policy.

## Data Availability

All data generated or analyzed during this study are available as part of the article, and no additional data sources are required.

## References

[1] Bandyopadhyay A , Bose S . Additive Manufacturing. CRC press; 2019. 10.1016/j.addma.2019.04.025 PMC6690615 31406683

[2] Zhang ZC , Li PL , Chu FT , Shen G . Influence of the three‐dimensional printing technique and printing layer thickness on model accuracy. J Orofac Orthop. 2019;80(4):194‐204. 31172199 10.1007/s00056-019-00180-y

[3] Revilla‐León M , Özcan M . Additive manufacturing technologies used for processing polymers: current status and potential application in prosthetic dentistry. J Prosthodont. 2019;28(2):146‐158. 29682823 10.1111/jopr.12801

[4] Revilla‐León M , Meyer MJ , Zandinejad A , Özcan M . Additive manufacturing technologies for processing zirconia in dental applications. Int J Comput Dent. 2020;23(1):27‐37. 32207459

[5] Jockusch J , Özcan M . Additive manufacturing of dental polymers: an overview on processes, materials and applications. Dent Mater J. 2020;39(3):345‐354. 32037387 10.4012/dmj.2019-123

[6] Sherman SL , Kadioglu O , Currier GF , Kierl JP , Li J . Accuracy of digital light processing printing of 3‐dimensional dental models. Am J Orthod Dentofacial Orthop. 2020;157(3):422‐428. 32115120 10.1016/j.ajodo.2019.10.012

[7] Alharbi N , Wismeijer D , Osman RB . Additive manufacturing techniques in prosthodontics: where do we currently stand? A critical review Int J Prosthodont. 2017;30(5):474‐484. 10.11607/ijp.5079 28750105

[8] Jindal P , Juneja M , Siena FL , Bajaj D , Breedon P . Mechanical and geometric properties of thermoformed and 3D printed clear dental aligners. Am J Orthod Dentofacial Orthop. 2019;156(5):694‐701. 31677678 10.1016/j.ajodo.2019.05.012

[9] Bhargav A , Sanjairaj V , Rosa V , Feng LW , Fuh YH J . Applications of additive manufacturing in dentistry: a review. J Biomed Mater Res B Appl Biomater. 2018;106(5):2058‐2064. 28736923 10.1002/jbm.b.33961

[10] Bukhari S , Goodacre BJ , AlHelal A , Kattadiyil MT , Richardson PM . Three‐dimensional printing in contemporary fixed prosthodontics: a technique article. J Prosthet Dent. 2018;119(4):530‐534. 28888410 10.1016/j.prosdent.2017.07.008

[11] Rungrojwittayakul O , Kan JY , Shiozaki K , et al. Accuracy of 3D printed models created by two Technologies of Printers with different designs of Model Base. J Prosthodont. 2020;29(2):124‐128. 31498957 10.1111/jopr.13107

[12] Park ME , Shin SY . Three‐dimensional comparative study on the accuracy and reproducibility of dental casts fabricated by 3D printers. J Prosthet Dent. 2018;119(5):861.e1‐861.e7. 10.1016/j.prosdent.2017.08.020 29475753

[13] Pillai S , Upadhyay A . Dental 3D‐printing: transferring art from the laboratories to the Clinics. Polymers. 2021;13(1):157. doi:10.3390/polym13010157 33406617 PMC7795531

[14] Standardization, I.O.f . Accuracy (trueness and precision) of measurement methods and results. Available from: https://www.iso.org/obp/ui/#iso:std:iso:5725:‐1:ed‐1:v1:en

[15] Dutton E , Ludlow M , Mennito A . The effect different substrates have on the trueness and precision of eight different intraoral scanners. J Esthet Restor Dent. 2020;32(2):204‐218. 31568660 10.1111/jerd.12528

[16] Gurpinar B , Tak O . Effect of pulp chamber depth on the accuracy of endocrown scans made with different intraoral scanners versus an industrial scanner: an in vitro study. J Prosthet Dent. 2022;127(3):430‐437. 33309210 10.1016/j.prosdent.2020.08.034

[17] Pérez M , Carou D , Rubio EM , Teti R . Current advances in additive manufacturing. Procedia CIRP. 2020;88:439‐444.

[18] Latham J , Ludlow M , Mennito A , Kelly A , Evans Z , Renne W . Effect of scan pattern on complete‐arch scans with 4 digital scanners. J Prosthet Dent. 2020;123(1):85‐95. 30982616 10.1016/j.prosdent.2019.02.008

[19] Chiu A , Chen YW , Hayashi J , Sadr A . Accuracy of CAD/CAM digital impressions with different intraoral scanner parameters. Sensors. 2020;20:4. 10.3390/s20041157 PMC7071446 32093174

[20] Rotar RN , Jivanescu A . Trueness and precision of two intraoral scanners: a comparative in vitro study. Scanning. 2019;2019:1289570. 31741697 10.1155/2019/1289570 PMC6854270

[21] Mădălina‐Georgiana, D. , Studiu Experimental Privind Variatiile Dimensionale Ale Obiectelor Printate 3d In Functie De Pozitia De Printare Cu Aplicatie In Protetica Dentara. Carol Davila University Library Archives; 2021.

[22] Zarone F , Ruggiero G , di Mauro MI , Spagnuolo G , Ferrari M , Sorrentino R . Accuracy of three impression materials on the totally edentulous maxilla: in vitro/in Silico comparative analysis. Materials (Basel). 2020;13:13. 10.3390/ma13030515 PMC7040790 31978974

[23] Formlabs . Model orientation best practices for SLA printing. Available from: https://support.formlabs.com/s/article/Model‐Orientation?language=en_US

[24] Alharbi N , Osman RB , Wismeijer D . Factors influencing the dimensional accuracy of 3D‐printed full‐coverage dental restorations using Stereolithography technology. Int J Prosthodont. 2016;29(5):503‐510. 27611757 10.11607/ijp.4835

[25] Shin S‐H , Lim JH , Kang YJ , Kim JH , Shim JS , Kim JE . Evaluation of the 3D printing accuracy of a dental model according to its internal structure and cross‐arch plate design: an in vitro study. Dent. Mater. 2020;13(23):5433. 10.3390/ma13235433 PMC7729473 33260676

[26] Song S , Zhang J , Liu M , Li F , Bai S . Effect of build orientation and layer thickness on manufacturing accuracy, printing time, and material consumption of 3D printed complete denture bases. J Dent. 2023;130:104435. 36693587 10.1016/j.jdent.2023.104435

[27] Hussein MO , Hussein LA . Trueness of 3D printed partial denture frameworks: build orientations and support structure density parameters. J adv Prosthodont. 2022;14(3):150‐161. 35855318 10.4047/jap.2022.14.3.150 PMC9259348

[28] Tahir N , Abduo J . An in vitro evaluation of the effect of 3d printing orientation on the accuracy of implant surgical templates fabricated by desktop printer. J Prosthodont. 2022;31(9):791‐798. 35067993 10.1111/jopr.13485

[29] Ko J , Bloomstein RD , Briss D , et al. Effect of build angle and layer height on the accuracy of 3‐dimensional printed dental models. Am J Orthod Dentofacial Orthop. 2021;160(3):451‐458.e2. 34456006 10.1016/j.ajodo.2020.11.039

[30] Gao H , Yang Z , Lin WS . The effect of build orientation on the dimensional accuracy of 3d‐printed mandibular complete dentures manufactured with a multijet 3d printer. J Prosthodont. 2021;30(8):684‐689. 33459450 10.1111/jopr.13330

[31] Shim JS , Kim JE , Jeong SH , Choi YJ , Ryu JJ . Printing accuracy, mechanical properties, surface characteristics, and microbial adhesion of 3D‐printed resins with various printing orientations. J Prosthet Dent. 2020;124(4):468‐475. 31810611 10.1016/j.prosdent.2019.05.034

[32] Nulty A . A comparison of trueness and precision of 12 3D printers used in dentistry. BDJ Open. 2022;8(1):14. 35618716 10.1038/s41405-022-00108-6 PMC9135705

[33] Morón‐Conejo B , López‐Vilagran J , Cáceres D , Berrendero S , Pradíes G . Accuracy of five different 3D printing workflows for dental models comparing industrial and dental desktop printers. Clin Oral Investig. 2023;27(6):2521‐2532. 10.1007/s00784-022-04809-y PMC10264508 36462040

